# Results for local control and functional outcome after linac-based image-guided stereotactic radiosurgery in 190 patients with vestibular schwannoma

**DOI:** 10.1093/jrr/rrt101

**Published:** 2013-08-26

**Authors:** Harun Badakhshi, Reinhold Graf, Dirk Böhmer, Michael Synowitz, Edzard Wiener, Volker Budach

**Affiliations:** 1 Departments for Radiation Oncology, Charité University Medicine Berlin, Augustenburger Platz 1, 13353 Berlin, Germany; 2 Department for Neurosurgery, Charité University Medicine Berlin, Augustenburger Platz 1, 13353 Berlin, Germany; 3 Institute for Neuroradiology, Charité University Medicine Berlin, Augustenburger Platz 1, 13353 Berlin, Germany

**Keywords:** vestibular schwannoma, acoustic neurinoma, stereotactic radiosurgery, image-guided intervention, local control

## Abstract

**Background:**

We assessed local control (LC) and functional outcome after linac-based stereotactic radiosurgery (SRS) for vestibular schwannoma (VS). *Methods* Between 1998 and 2008, 190 patients with VS were treated with SRS. All patients had tumors <2 cm diameter. Patients received 13.5 Gy prescribed to the 80th isodose at the tumor margin. The primary endpoint was LC. Secondary endpoints were symptomatic control and morbidity. *Results* Median follow-up was 40 months. LC was achieved in 88% of patients. There were no acute reactions exceeding Grade I. Trigeminal nerve dysfunction was present in 21.6% (*n* = 41) prior to SRS. After treatment, 85% (*n* = 155) had no change, 4.4,% (*n* = 8) had a relief of symptoms, 10.4% (*n* = 19) had new symptoms. Facial nerve dysfunction was present in some patients prior to treatment, e.g. paresis (12.6%; *n =* 24) and dysgeusia (0.5%; *n* = 1). After treatment 1.1% (*n* = 2) reported improvement and 6.1% (*n* = 11) experienced new symptoms. Hearing problems before SRS were present in 69.5% of patients (*n* = 132). After treatment, 62.6% (*n* = 144) had no change, 10.4% (*n* = 19) experienced improvement and 26.9% (*n =* 49) became hearing impaired. *Conclusion* This series of SRS for small VS provided similar LC rates to microsurgery; thus, it is effective as a non-invasive, image-guided procedure. The functional outcomes observed indicate the safety and effectiveness of linac-based SRS. Patients may now be informed of the clinical equivalence of SRS to microsurgery.

## INTRODUCTION

The clinical management of patients with small-sized vestibular schwannomas (VS) is still an area of debate. The choice is between watchful waiting, microsurgery and stereotactic radiosurgery (SRS). The option of watchful waiting might be an adequate approach for neurologically marginally-hampered patients [1]. Traditionally, microsurgery has been the mainstay of treatment of VS in recent decades and offers excellent tumor control [2]. SRS, implemented by Leksell has now become an additional tool [3]. SRS can be delivered modern linear accelerators with stereotactic tools [4–7]. This study focuses on the results of long-term outcomes with respect to the feasibility, safety and effectiveness of SRS for VS.

## MATERIALS AND METHODS

### Study design

Between 1998 and 2008, 190 patients with small-sized (<2 cm, median 1.2 cm, range, 0.6–2 cm) VS underwent linac-based SRS of 13.5 Gy (*n* = 190). After receiving the Institutional Review Board's agreement, we obtained informed consent from all patients. A prospective database was established. Most patients (82%) had regular follow-up visits including MRI twelve weeks after SRS and at three- to six-month intervals. There was a minimum follow-up period of five years for 75% of patients. Two-thirds of those had regular MRIs of the brain until five years after treatment. An update of missing clinical data was compiled by approaching patients and general practitioners. To assess the treatment efficacy, volumetric measurements of the repetitive MRIs were done in 72.3% (*n* = 182) of all cases. Primary outcome measured LC rate, defined as the case of stable disease or tumor regression assessed by means of cranial MRI at the latest follow-up. Moreover, disease-related symptoms before and after treatment were reported according a validated toxicity score as follows: tinnitus, dizziness, dysfunction of trigeminal and facial nerves, and gradual loss of hearing. A vast majority of patients had no audiometry by a quantitative method; this is a clear limitation of the study. We interviewed patients about their hearing quality in quotidian life, thus investigating how serviceable their hearing really was. We trusted the steadiness of patient's subjective statements in regard to the hearing function.

### Patient characteristics

We analyzed 102 females and 88 males. The median age was 59 years. Of the 190 patients, 156 had primary diagnoses and 34 had recurrences. In three patients VS was associated with neurofibromatosis (NF-2). The median follow-up was 40 months; 46.8% of the patients had a follow-up after >36 months, 21.2% after >60 months. We observed good clinical practice, and data analyses were only executed after a positive vote by the local ethics committee and the informed consent of the patients (Table 1).

**Table 1.** Baseline patient and tumor variables for 190 patients treated for vestibular schwannoma with SRS

Parameter	Characteristic	No. of patients (% of total)
Gender	Male	88 (46.3%)
	Female	102 (53.7%)
Genetic predisposition	Sporadic	189 (99.5%)
	NF-2	1 (0.5%)
Hearing difficulties before SRS	Yes	132 (69.5%)
Trigeminal dysfunction before SRS	Pain, dysesthesia	41 (21.6%)
Prior surgery	Yes	34 (17.9%)
Side	Left	92 (48.4%)
	Right	98 (51.6%)

### Technical set-up

From 1998–2003, patients underwent ‘sharp’ fixation using a stereotactic head ring supplemented with an oral bite plate to ensure placement reproducibility. A conventional 6-MV linac (Varian^®^ USA) equipped with an add-on micro-multileaf collimator (mMLC) (BrainLab^®^ Co., Germany) was used. The target coordinators for SRS were set by a laser-based stereotactic localizer, which could be adjusted with six degrees of freedom (6DOF). This hardware and software set-up allowed the delivery of shaped beams. In 2004 we started using a Novalis^®^ (BrainLab^®^) with built-in MLC, beam-shaping capability and the option of online image guidance of the treatment delivery. This significant development in technology enabled us to register and verify the target localization and to carry out real-time adaptation of the therapy set-up. The mechanical accuracy was 0.6 mm. The new infrastructure enabled transformation of the set-up of immobilization into a less invasive device in combination with the aforementioned stereoscopic image guidance and 6DOF table corrections. From this point on we used a Novalis ExacTrac^®^ image-guided frameless system, which enabled us to image the patient in any couch position using a frameless positioning array. For the entire period reported on here, we performed image fusion using MRI and planning CT. The target volumes and organs at risk were delineated on each slice of MRI and CT using the 3D treatment-planning system Brainscan^®^ (Brain Lab AG, Germany). The gross tumor volume (GTV) was defined as the area of contrast enhancement on T1-weighted MRI images, and the planning target volume (PTV) included a 1-mm safety margin to allow for possible patient positioning errors.

The dose was prescribed to a reference point, which was the isocenter (or the center of GTV), though 100% was not the maximum dose but the dose at the aforementioned reference point.

**Fig. 1.**
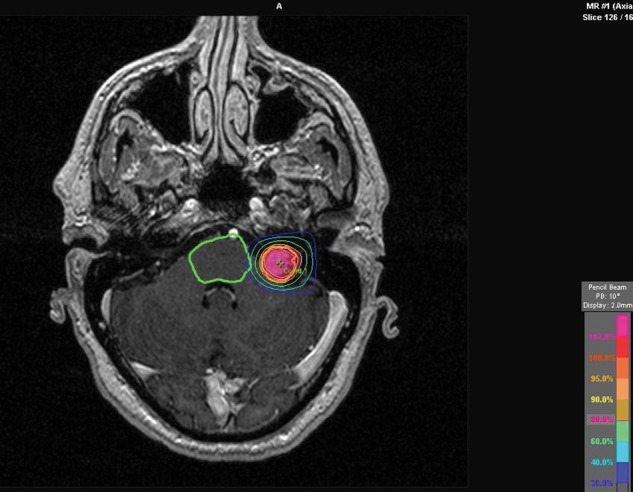
MRI-based target definition, 100% dose at reference point.

Patients received 13.5 Gy prescribed to the 80th isodose at the tumor margin (Fig. 1).

### Statistics

LC and hearing preservation probabilities after radiotherapy were calculated using the Kaplan–Meier method. For intergroup differences, the Student's *t*-test and the log-rank test were applied. All statistical analyses were performed using IBM SPSS Statistics 19 (New York, USA).

## RESULTS

### Local tumor control

LC was defined as lack of progression or tumor regression on MRIs at follow-up visits. Of the 190 patients, 15 (7.8%) were lost to follow-up, leaving 92.1% (*n* = 175) of patients with complete clinical data for the analysis.

**Fig. 2.**
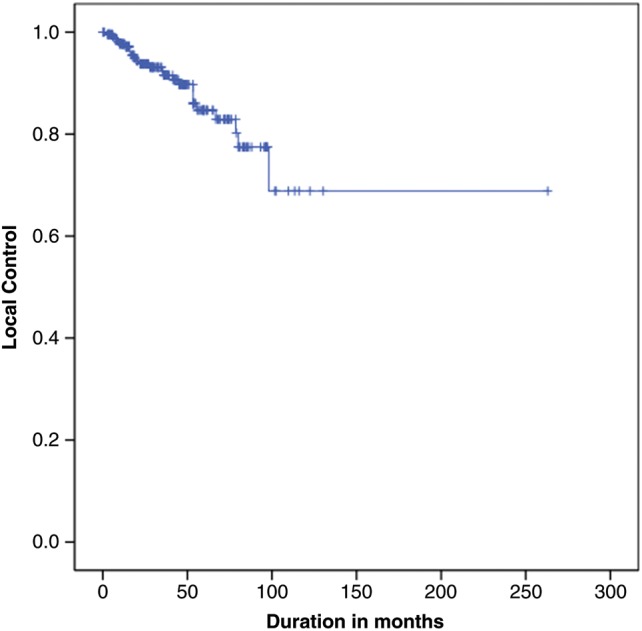
Local control (LC) in months.

For 88.0% (*n* = 154) a crude LC rate was achieved. Progression was observed in 11.1% (*n* = 21) of all cases. At 3 years, an LC rate of 92%, and at 5 years an LC rate of 68%, was achieved. Volumetric analysis was done using MRI examinations during follow-up. Only 1.3% (*n* = 3) of all lesions showed a volumetric progression to such an extent that surgical treatment was required. Thus, 98.7% of the patients needed no additional surgical intervention (Fig. 2).

### Morbidity

Acute side effects during or shortly after treatment (period of 90 days) were rare. No Grade II or greater toxicity occurred. Glucocorticoids had to be prescribed because of headache and nausea during and after treatment in 36 (18.6%) patients, the majority (*n =* 28) of this subgroup had been in need for a short time of some weeks.

### Functional outcome

#### Tinnitus

Tinnitus was documented in 59% of all patients (*n* = 112) prior to treatment. Mild, moderate and severe symptoms were reported in 25.3% (*n* = 48), 30.5% (*n* = 58) and 3.2% (*n* = 6) of all cases, respectively. After treatment, no change in symptoms was reported in 73.1% of patients (*n* = 133), improvement was reported in 14.4% (*n* = 26), and impairment was reported in 12.6% (*n* = 23) (Table 2).

**Table 2.** Functional outcome with respect to tinnitus before and after treatment

	Number of patients
Prior to SRS	59% (*n* = 112)
Impairment of symptoms after SRS	12.6% (*n* = 23)
Relief of symptoms after SRS	14.4% (*n* = 26)

#### Dizziness

Dizziness was reported in 42.1% (*n* = 119) prior to radiation. Mild, moderate and severe symptoms were reported in 20.5% (*n* = 39), 34.7% (*n* = 66) and 7.4% (*n* = 14) of all cases, respectively. After treatment, no change in symptoms was reported in 75.1% of patients (*n* = 104), improvement was reported in 28.6% (*n* = 52), and impairment was reported in 14.3% (*n* = 26) (Table 3).

**Table 3.** Functional outcome with respect to dizziness before and after treatment

	Number of patients
Prior to SRS	42.1% (*n* = 119)
Impairment of symptoms after SRS	14.3% (*n* = 26)
Relief of symptoms after SRS	28.6% (*n* = 52)

#### Trigeminal nerve dysfunction

Trigeminal nerve dysfunction was reported prior to therapy in 21.6% (*n* = 41) of cases. After treatment, 85% (*n* = 155) had no change, 4.4% (*n* = 8) of formerly affected patients had a relief of symptoms, 10.4% (*n* = 19) had new onset of symptoms such as pain and dysesthesia (Table 4).

**Table 4.** Functional outcome with respect to trigeminal neuropathy before and after treatment

	Number of patients
Prior to SRS	21.6% (*n* = 41)
Impairment of symptoms after SRS	10.4% (*n* = 19)
Relief of symptoms after SRS	4.4,% (*n* = 8)

#### Facial nerve dysfunction

Facial nerve dysfunction was documented prior to treatment, e.g. paresis 12.6% (*n* = 24) and dysgeusia (0.5%; *n* = 1). After treatment, improvement was reported in 1.1% (*n* = 2), 6.1% (*n* = 11) experienced new symptoms (Table 5) and 92.8% had no symptoms.

**Table 5.** Functional outcome with respect to facial neuropathy before and after treatment

	Number of patients
Prior to SRS	12.6% (*n* = 24)
Impairment of symptoms after SRS	6.1% (*n* = 11)
Relief of symptoms after SRS	1.1% (*n* = 2)

#### Hearing problems

Hearing problems before SRS were present in 69.5% (*n* = 132). After treatment, 62.6% (*n* = 144) had no change, 10.4% (*n* = 19) experienced improvement, and 26.9% (*n* = 49) developed impaired hearing (Table 6)[Table RRT101TB7].

**Table 6.** Functional outcome with respect to hearing difficulties before and after treatment

	Number of patients
Prior to SRS	69.5% (*n* = 132)
New onset of symptoms after SRS	26.9% (*n* = 49)
Relief of symptoms after SRS	10.4% (*n* = 19)

**Table 7. RRT101TB7:** Outcome of image-guided linac-based SRS with respect to local control and functions

Study	*n*	Dose in Gy	Control rate (%)
Suh	29	16	94 @ 5 year
Foote	149	14	87 @ 5 year
Spiegelmann	48	14	98 @ 3 year
Okunaga	46	14	100 @ 5 year
Roos	65	13	95 @ 4 year
Combs	26	13	91 @ 5 year
Rutten	26	12	95 @ 5 year
Friedman	390	13	90 @ 5 year
Hsu	75	15	92 @ 5 year
Badakhshi	190	13.5	88 @ 3 year

## DISCUSSION

Treatment options for VS include surgery and SRS. Watchful waiting is an accepted strategy. Surgery by expert surgeons has an LC rate greater than 95% [2]. SRS is achieving similar LC rates according to a recently published meta-analysis of 37 case series, comprising 3,677 patients. The overall LC rate observed was 92.2% (95%, CI: 90.4–93.7%) [8].

The main limitation of this study was the retrospective analysis, although it was based on a prospective database. Another major confounding factor in this analysis was the fact that data acquisition, in terms of disease and treatment-related functional problems, was done subjectively by the patient or the physician and not objectively. The information gathered on functionalities, e.g. hearing preservation, as a relevant parameter for patient quality of life, was not based on objective auditory analyses. These factors are major drawbacks. Additionally, it is very important to note that the vast majority (ca. 40%) of patients experienced transient tumor expansion during the first 4 years after SRS. Therefore, the LC rate shown in this study might be confounded by transient volumetric change after SRS. The transient volumetric change can cause worsening of symptoms that usually resolves once tumors enter the shrinking phase.

The vast majority of publications are based on Gamma knife applications [9]. In order to critically evaluate our results with linac-based SRS techniques, we extracted those papers reporting only linac-based SRS. Suh and colleagues reported on 29 patients, 12 of whom underwent surgery before SRS. The median tumor volume reported was 2.1 cm^3^, and LC was achieved in 28 of 29 patients. This finding translated into an actuarial 5-year LC rate of 94% [10]. Foote and colleagues reported on the results of SRS in 149 VS cases, of whom 28% had had prior surgery and the mean tumor volume was 4.8 cm^3^. A mean dose of 14 Gy (range, 10–22.5 Gy) was given to the 80% isodose. After a median follow-up of 34 months, LC was achieved in 93% of patients; the actuarial LC rate at five years was 87% (95% CI, 76–98) [11]. This data corresponds well with the present study, although our dose prescription was 13.5 Gy at the 80% isodose. Spiegelmann *et al*. published data on 48 cases, with a median tumor diameter of 20 mm. Lesions up to 16 mm in size received a maximum of 14 Gy encompassing the PTV; larger tumors were treated with a minimum dose of 11 Gy. After a median follow-up of 32 months, LC was achieved in 98% of patients, which is comparable to the results of surgical series [12]. Okunaga *et al*. published results for 46 patients; 26.1% of the patients had had prior surgery and a median tumor volume of 2.29 cm^3^ (0.4–7 cm^3^) was reported. The prescribed dose was 14 Gy. Median follow-up was 56.5 months. An LC of 73.8% was observed in all patients followed up for >1 year, and an LC of 100% was observed in the 18 patients followed for > 5 years [13]. Roos *et al*. reported on 65 patients with VS with a median diameter of 22 mm, treated with SRS of 13 Gy. The median follow-up period was 48 months, and LC was observed in 95% of patients [14]. Combs published data on 26 patients treated with a dose of 13 Gy. The actuarial LC rates at 5 and 10 years for all patients were 91% in both cases [15]. Rutten *et al*. have reported on SRS for 26 patients with VS with a median size of 18 mm, a dose of 12 Gy, and a median follow-up of 49 months. The 5-year LC rate reported was 95% [16]. The largest series to date included 390 cases and reported an LC rate of 98% at 2 years and 90% at 5 years [17].

The above studies show clear evidence for the use of SRS with a dose of 12–14 Gy resulting in 5- and 10-year LC rates of ∼ 90% [18].

Early and early-delayed toxicity can be defined as sequelae and complications within weeks to months after SRS. These include headache, local erythema of the skin, alopecia of in-field treatment areas, and fatigue. We experienced no cases of severe acute toxicity among our patients.

Functional outcome with respect to nerve function is a difficult issue to judge clearly because of the range of reported data that could be affected by different cofactors. Combs reported 2.5% new cases of tinnitus after SRS. Most cranial neuropathies following radiosurgery are mild, transient and commonly present as late-delayed complications. The mean latency of trigeminal and facial neuropathies after SRS for VS has been noted to be ∼ 7 months. Ito *et al*. report a similar latency of 4–5 months for the onset of cranial neuropathies [19].

Trigeminal and facial neuropathy has been reported in 8% and 5%, respectively, in Comb's paper [15], and in 8–18% in Spiegelmann's publication [12]. Our data seem to be in the range of potential toxicity with respect to hearing (and therefore quality of life). However, for other symptoms, data from a recent meta-analysis indicates a clear benefit of SRS [20] in comparison with surgery, which is in line with our results.

## CONCLUSION

This study of a very large patient cohort treated with image-guided linac-based SRS yielded a high rate of LC, favorably comparable with microsurgery. Individual counseling of patients by an interdisciplinary team is desirable in order to provide adequate information for decision-making about treatment. Taking into account the above-mentioned confounding factors, the results of the present study provide valuable data on LC and functionality of cranial nerves for VS treated with linac-based SRS.

## FUNDING

The authors all confirm that there has been no funding to support the research for this paper.
